# Dr. Duke on the Cerebral Affections of Infancy and Childhood

**Published:** 1849-01-01

**Authors:** 


ART. III.—*On the Cerebral Affections of Infancy and Childhood.* By VALENTINE DUKE, ESQ., M.D. (Being the Council Prize Essay, awarded at the Annual Meeting of the Provincial Medical and Surgical Association, held at Bath.) 1848.

WE feel anxious to place before our readers the most recent observations in connexion with the physiology and pathology of the nervous system. We therefore have selected, for analytical review, an Essay, just published in the "*Transactions of the Provincial Medical and Surgical Association,*" by Dr. Duke, on the cerebral diseases of children.

This class of affections is deserving of greater consideration than it has hitherto received from the hands of those competent by practical experience to enlighten the profession on the frequently obscure points connected with the cerebral diseases of early life. These affections are common during the periods of infancy and childhood. They are often most fatal,—most insidious in their progress,—most obscure in their origin,—most difficult of diagnosis, and, alas! very frequently setting at defiance all the resources of our art. Owing to the impossibility of obtaining accurate information from the patients themselves, the physician is required to exercise great tact and discrimination in the detection of the diseases of the brain and nervous system, with the view of recognising at the earliest period the first symptoms of disease; for, at this stage, it may be possible to prevent the brain and its appendages from becoming organically affected. None but those who have had some practical acquaintance with the management of the diseases of early life, are competent to appreciate the difficulties which the physician has often to encounter in the treatment of the cerebral affections of children. The medical man is not often called in "until the time has passed, when the early observation of symptoms might have afforded greater facilities for drawing inferences. Unfortunately, it happens, often too, that cerebral affections are so insidious in their approach, so masked by some prominent, remote, sympathetic affection, or so little characterized by any very decided pathognomonic symptoms, that they are overlooked by the friends in the early stages, and the doctor is called in, only to be able to confirm the apprehension that has been accidentally excited for the safety of the head. The child may have been astray for some time, and have lost flesh,—but it was only teething, and has often been so before. True, it has had vomiting, but then the food disagreed; or if it be an infant suckling, the mother has been anxious, and has lost her rest—enough to cause that. There is a ready and familiar way to account for every symptom, but the lurking disease is overlooked, or not suspected. The supervention of convulsions, screaming, severe head-ache, or perhaps, sudden coma, arouses anxiety, and directs attention to the head, and then, too often, irremediable mischief has been done.

In relation to the medical literature connected with this department of practical medicine, Dr. Duke observes, that "until the work of Dr. Whytt (published in Edinburgh) appeared, this subject had not received any real scientific attention." The writings of Drs. Fothergill, Watson, Dobson, Rush (of Philadelphia), Quin (of Dublin), Cheyne, West, Wiltshire, Marshall Hall, are referred to. Dr. Duke makes no mention of Mr. W. C. Dendy's valuable monograph, "*On the Cerebral Diseases of Children, particularly in reference to their early Manifestations and Treatment*," which appeared in an early number of the "Psychological Journal."

The author commences his treatise with the subject of "Congestion of the Brain." This affection, he says, is of rare occurrence in early life. It is generally the result of a neglected state of the bowels, exposure to cold, and causes impeding the return of blood to the head. He has known this affection terminate fatally in twenty-four hours—the result of sudden passion in the nurse. He believes congestion of the brain is occasionally the consequence of mothers, whilst suckling, freely indulging in spirituous and fermented liquors. It also occurs during the course of the exanthemata and hooping-cough. The symptoms of the disease, as recorded by Maunsell and Evanson, are, stupidity and heaviness, "the head looking full, and being perhaps hotter than usual, the veins distended and dark coloured, the countenance livid, and the pulse slow, or irregular; the pupils are usually dilated, and the eyes looking vacant; a permanently elevated and convex condition of the fontanelles leaves no doubt of its existence. If unrelieved, these symptoms may be followed by effusion, and death take place in twenty-four hours."

The treatment consists (after removing the cause) in cold applications to the head, which, with perhaps the general warm bath, and freely acting on the bowels, will generally be sufficient; but if the lividity of countenance, or the nature of the breathing, denotes danger, we must apply leeches.

Blisters will also be found useful, and both these remedies are recommended to be applied to the extremities, rather than the head. The nape of the neck should be selected as the part best suited for blistering. Tonics and stimulants may be necessary to prevent a recurrence of the symptoms of congestion.

*Irritation, or Erethism of the Brain,* is generally associated with dentition. There is increased irritability of the sensorium, and susceptibility to impressions. The child, startled by the slightest noise and light, is uneasy and fretful. The eyelids are generally closed; the flexion of the thumb on the palm of the hand is a peculiar symptom. The child is watchful, and gets little sleep. It is distinguished from congestion of the brain by the absence of stupor, and the tendency to coma. Hydrocephalus is the frequent sequel of this condition of the brain. The treatment consists in allaying irritability, procuring sleep, and supporting the strength by light stimulating nourishment. Evacuate the bowels by enemata and mild aperients—apply cold to the head, and warmth to the feet. The latter object is effected best by means of flannel, wrung out of hot water, rolled round the feet and legs—this again being wrapped up in a warm, dry piece of flannel, or small blanket. Change of air sometimes acts like a charm. The state of the gums must, of course, be attended to, and if necessary the gum lancet ought to be freely used.

*The Hydrencephaloid Disease* is divided into two stages,—the first, that of irritability; the second, of torpor. In the former there is a feeble attempt at reaction; in the latter, the powers appear to be prostrate. Dr. Duke observes, "If we trace the history of the case, we shall generally find that there has been some considerable evacuation, either loss of blood in the cure of some other affection, or long existing diarrhœa. The infant at first is irritable and peevish; he starts upon being touched, and is over-sensitive. There is sighing and moaning during sleep, and sometimes screaming. Here are symptoms, some of which are present in the erethism of the brain, and some in hydrocephalus; and it will require the closest attention to the history of the case to enable us to discriminate between them. As the disease advances, the exhaustion is more apparent; the countenance becomes pale, and the cheeks cool; the eyelids are half closed, the eyes fixed, and unattracted by any object placed before them; the pupils unmoved by light; vomiting is sometimes present, and the bowels are rather free than constipated, though the evacuations are unhealthy. You must meet these symptoms by supporting the system, administering gentle stimulants, and such moderate doses of opium as may allay irritability, or check diarrhœa, if present. Dover's powder is a very manageable form in which to use this medicine, and the starch enema, with opium, will be generally effectual when diarrhœa exists. If vomiting be present, we must try to allay it by administering small quantities of nourishment, as chicken-broth; or, if the exhaustion be very great, by stimulants, as wine, or even brandy. A small blister, for an hour, over the stomach, will often materially assist in checking vomiting. If there be great coldness of the surface, we may use the warm bath for a short time; and the water may be made stimulating, by the addition of some mustard. After the symptoms have been subdued, tonics must be administered to prevent relapse, and, as before-mentioned, change of air is most desirable. Should we, by oversight or mistake of its real character, treat this case by continued depletion, our patient would assuredly sink rapidly, and die comatose, or convulsed.

The application of cold to the head, so useful in most of the cerebral affections of infancy, would here be badly borne.

Should the symptoms not be relieved, the child may die seemingly exhausted, and examination would very likely show considerable serous effusion in the ventricles; but then this would have been more of a passive nature, than the result of acute inflammation.

Symptoms of exhaustion are frequently seen in children who are imperfectly nourished, either on account of deficient supply, or the bad quality of the nurse's milk. These matters must be closely inquired into by the physician, and his treatment regulated accordingly. The change of a nurse, under such circumstances, is imperatively called for.

Great attention should also be paid to the nourishment a child receives after being weaned, especially if that process has been suddenly carried into effect, from any necessary cause. The assimilating powers of the digestive apparatus are weak, and disease of the brain may result from deficient nutriment.

*Convulsions of Children.*—On this subject, Dr. Duke broaches nothing novel. This is a common affection in early life, and is often dependent upon some irritation existing in the primæ viæ, or from difficult teething. Delicate children are more liable to this disease. When the robust are attacked, the danger to life is greater. Dr. West observes that convulsions in infancy and childhood appear to take the place of delirium in adult life. In the treatment of this class of affections, we shall "generally proceed safely in ordering cooling applications to the head, having the bowels well freed, and directing the use of the tepid or warm bath; sprinkling cold water in the face is a means also recommended; I have seen it shorten and prevent the seizure. When some distinctive symptoms are present, localizing the cause, we can apply ourselves more particularly to their removal. Carminatives, with turpentine and assafœtida enemata, will procure the discharge of flatus, generally followed by great relief.

"Lancing the gums should never be neglected; it will often induce sound sleep, and hinder a return of the attack. Sometimes, especially where there is a tendency to somnolence or coma after the seizure, the application of a couple of leeches will considerably lessen the liability to a recurrence. In such a case, we must be very cautious in the use of cold to the head."

*Acute Meningitis* is a most rapidly fatal disease. Fortunately, it is of rare occurrence. Dr. Duke considers it a distinct affection. It generally comes on suddenly, and attacks children previously healthy and robust. This disease is often the consequence of exposure, for a lengthened period, to the rays of the sun, blows on the head, the sudden suppression of purulent discharge from the ear, the repercussion of cutaneous affections of the scalp. This affection is divided into the phrenitic and convulsive forms. We do not see the practical value of this division, because the two affections are so often blended together. Our author says the phrenitic form occurs more frequently in children of four years old, and upwards, who have begun to exercise actively the reasoning powers (?) ; the latter, in younger infants.

"The convulsive form usually commences suddenly, by an attack of convulsions, general or partial. There is febrile disturbance, but not so great as in the phrenitic variety; vomiting and constipation are not so usually observed. The convulsions are repeated at short intervals; and between times, the child remains either very much agitated or comatose, with the pupils mostly contracted, and very likely there is squinting; sometimes we have hemiplegia.

"A rigor, followed by fever and vomiting, with head-ache, intolerance of light and noise, quick pulse, hot and burning skin" generally accompanies the phrenitic form. The child is restless, and answers questions abruptly. "The intellect becomes soon engaged, and, as the disease advances, the symptoms indicative of derangement of the nervous system become more marked; the head-ache is intense, with frowning; delirium, of a violent character, is present, alternating with stupor. Convulsions now occur, and there is strabismus, with contracted pupil. The child lies with his eyes closed, and face averted from the light; he often starts and screams with the violent pain, which is so severe as quite to deprive him of sleep. The respiration and circulation are also much affected; the former is hurried and irregular, presenting that character so well known in fever, as cerebral breathing. The pulse is very quick, and generally strong and full, but variable. The face is alternately flushed and pale, with an aspect, wild at times, at others, expressive of great suffering. The skin is burning hot and dry. The digestive system also sympathizes. The tongue is red and dry; vomiting of bilious matter incessant, and the thirst urgent. The excretions from the bowels are scanty, and the urine high-coloured and diminished in quantity. If the disease be not arrested by treatment, the convulsions become more frequent and severe, with grinding of the teeth, strabismus, dilated pupil, and coma; where it is protracted, hemiplegia is sometimes present, and occasionally spasms of the muscles of the arms and legs; these latter, more particularly, when there has been an extension to the spine of the diseased action."

In the early stage of this disease it is not easily confounded with congestion of the brain. The acute pain, quick pulse, burning skin, the frowning, and grinding of the teeth, are the symptoms which will enable the practitioner to distinguish it from the former affection. We must be careful in not mistaking acute meningitis for the pneumonia of children, which is often ushered in by vomiting and convulsions. We ought also to recollect that symptoms of hydrocephalus sometimes are manifested in the last stages of pneumonia, the result of hyperæmia of the brain. The convulsions from teething, worms, &c., are less frequent, the attacks intermit, the pulse and respiration are but little accelerated. The patient does not exhibit that peculiar frown, and gives no indications of pain in the head. In acute meningitis, no marked remissions take place. Convulsions are sometimes the consequence of hæmorrhage into the arachnoid membrane. This is a rare disease. M. Legendre says, "Children attacked with meningeal apoplexy exhibit contractions of the fingers and toes, a symptom not observed in meningitis."

This affection is sometimes confounded with otitis. In the latter disease, the child gives evidence of acute local suffering, and generally "lies on the affected side, and presses it (the head) against the pillow, or keeps the hand to the ear." In the early stage, bleeding locally and generally, purgatives, with fœtid and turpentine enemata, are the principal remedies, modified according to the age of the patient. The gums should be effectually lanced. In severe cases, where the symptoms show no desire to remit, the patient should be placed under the influence of nauseating doses of the tartrate of antimony. With these remedies should be conjoined the warm bath, cold to the head, purgatives, calomel to salivation, fomentations, and sinapisms to the lower extremities.

Dr. Duke recommends also "the early shaving of the head, and application of counter-irritants to the scalp, more especially when we have reason to suspect that the retrocession of a cutaneous affection may have had any share in inducing the disease. Tartar-emetic ointment, with croton oil, will generally be sufficient for this purpose. Before having recourse to this latter means, in cases where we do not suppose that the disappearance of an eruption has had anything to do in producing the meningitis, the application of cold should be long and perseveringly tried. A thin towel, frequently wetted and kept upon the head, seems as good a method of using this as we can select. Ice in bladders is also recommended; and a method of slow irrigation, by means of a thread, conducting cold water from a vessel placed over the child's head, is recommended by M. Rilliet. We have had the child's head held over the side of the bed, and cold water poured in a stream from the spout of a tea-kettle, for several minutes, with decided benefit.

In our attempt to introduce mercury into the system, we need not fear to combine it with small quantities of opium. The tendency to coma is not so great as to be increased by the latter to any alarming extent. Small doses of Dover's powder will be found very useful in allaying irritation, and procuring sleep.

*Tubercular Meningitis*, or *Hydrocephalus Acutus.*—This is a disease of every stage of early life. It is frequently preceded and accompanied by derangement of the digestive functions, and often dependent upon, or associated with, the irritation of dentition. There are no symptoms which unequivocally point out the occurrence of effusion into the ventricles,—those cases which have proved fatal exhibiting the worst symptoms, severe convulsions, strabismus, coma, &c., often, upon dissection, furnishing the least quantity of watery effusion, and *vice versa.* This disease has almost always tubercular origin, and is frequently complicated with tubercular deposit elsewhere than in the brain. The liver is said to be the organ mostly at fault in this disease. Dr. Cheyne's* description of the incubation of the disease is so true to nature, that we offer no apology for copying it *in extenso*:—

"We find that before any characteristic signs appear, the patient, for some days, or even weeks, has complained of pains in his head or belly, while at the same time he has been slightly feverish, dull, vertiginous, sallow, without appetite, or perhaps with an increased or capricious one, and with a considerable disorder in all the functions of the abdominal viscera. In some instances a dragging of one of the legs has been observed, which has led to a fruitless examination of the hip-joint and spine. In others, a very painful crick in the neck has taken place, as the first symptom of disease. These complaints arise gradually, and the child's friends are not awakened to a sense of danger until, advancing a step farther, the commencement of a specific disease has more dis-

* Of Dublin.

tinctly shown itself. The dulness and severe pains are now accompanied with vomiting, usually upon getting up in the morning, or after the child has begun to stir about; yet even this symptom is often disregarded until the second or third time of its recurrence, and the disease has made considerable progress before the illness of the patient is suspected to arise from a disordered condition of the brain. When the attention is more particularly excited by these symptoms, the head-ache, or pain in the forehead, will be observed returning at shorter intervals. The child often affectingly complains of his head; he sighs frequently, is dull, his head requires to be supported, he complains of weariness in his eyes, the pupils appear unusually contracted, and he has an aversion to light; but in the dark, he sometimes fancies he sees flashes of light. His tongue is white, and his belly generally costive. The stools at first are of the colour of clay, but as the disease advances, they become of a gelatinous consistence, of a dark-green colour, and they have a peculiar smell, not unlike the smell of the breath in the beginning of some of the exanthemata. The pulse becomes quick, and at peculiar times these symptoms are attended with febrile heat and irritability, and the child complains, not of head-ache only, but of pains in different parts of the body, which are sometimes exceedingly acute. At one time, he will complain of pains in his limbs, at another of pain in his breast, or in the nape of the neck, very often in his bowels; but before his friends can make any preparation to relieve him, the pain ceases, or is transferred to some other part; at another time, he will lie long on his mother's knee, restless and whining, from dull rheumatic pains. These disorders cannot last long without impairing the child's strength; accordingly, in ten days or a fortnight, the period usually occupied by the first stage of this attack, his appearance is altered, his manner becomes peevish, his hand tremulous, and his gait tottering.

"In the second variety, which is less frequent, the disease runs a more rapid course. After the child has been in a drooping state for a short time, which, although it sometimes may escape observation, is generally recollected, there is a sudden change to a fever, attended, even from the first, with a great degree of pyrexia, with frequent, but short and irregular remissions, flushing, severe head-ache, tenderness all over the abdomen, and increased sensibility, with, sometimes, brilliancy of the eyes. It is said to be often difficult immediately to distinguish hydrocephalus from fever, and this is the form in which there is the greatest resemblance between the two diseases; but we are led to suspect some deeply-seated evil, from his frantic screams and complaints of his head and belly, alternating with stupor, or rather lowness, and unwillingness to be roused; and we are struck with the great irritability of the stomach, which exists in a degree beyond what we generally find it in the fevers of this country, retching and vomiting being brought on by a change of posture, and certainly by every attempt to sit up in bed; and the disordered state of the bowels which attends this irritability of the stomach is also remarkable; and when at any time the child has a little respite from the violence of these symptoms, we find our suspicions confirmed by his look, for when the features do not express pain or terror, there is not unfrequently a vacancy of look, the eyes being set with an expression of dejection which is peculiar to certain diseases of the brain."

He again says,—"When hydrocephalus arises during an indifferent state of health, as for example, after there had existed a scrofulous disease which has subsided, or where, from predisposition and from the anomalism of symptoms, some such disease might have been expected; or where the child has had some epidemic disease, as measles or scarlatina, from which he has not perfectly recovered, or regained sound health; the attack is sometimes made with all the violence which I have described as distinguishing the second form. When, again, the attack comes on as the sequel of an acute disease,—as, for instance, remittent fever, hooping-cough, dentition,—the child almost imperceptibly slips into hydrocephalus. There are scarcely any of the symptoms of the early stages, and paralysis and convulsions are sometimes the first indications of the new disorder."

This affection is often, perhaps almost always, associated with a strumous diathesis. Whatever causes tend to bring into activity latent scrofula, will induce hydrocephalus. Hence, we frequently find this disease among the children of the poor. Unwholesome and insufficient food, filthy and ill-ventilated houses, inattention to the state of the digestive apparatus, are the too frequent causes of hydrocephalus in humble life. Under better circumstances, the disease is often developed as the result of over-taxing the brain and intellect, stuffing the poor child with rich and improper food, and subjecting it to irregularities of diet, bad air, late hours, excitement of mind, &c. This disease is often the sequelæ of the exanthemata, measles, scarlatina, and it frequently developes itself during attacks of remittent fever. All debilitating causes and sources of general irritation may produce this formidable affection. It is important to detect the earliest symptoms of hydrocephalic disease. Dr. Duke considers, seriatim, the derangements of the *nervous*, *circulatory*, *respiratory*, and *digestive* systems.

In cases where we have reason to believe that hydrocephalus has commenced its formidable train of symptoms, Dr. Duke says we shall detect, in the first instance, as an affection of the nervous system, restlessness and irritability about the child's manner,—altered temper, an uneasy, whining, and distressed look,—anxiety of countenance, heavy eyes,—pupils contracted, aversion to light and noise,—an expression of sudden alarm,—taking no pleasure in his former amusements. He has pain in different parts of the body,—particularly of the head, causing the little patient to scream out loud. The pain in the head is sometimes absent, and is occasionally only noticed at night, when the child will suddenly awake in a fright, and give a scream, complaining of pain in the head.

*The Circulating System.*—In this disease, we have early evidence of increased vascular action. The pulse and carotids give evidence of this. The former is sometimes wiry and harsh.

*The Respiratory System* is also in rotation affected. There is a great tendency to sigh, and the patient is sometimes troubled with a short, dry and hacking cough.

*The Digestive Functions* are often seriously at fault. The patient may, at an early period of the attack, have great irritability of stomach; vomiting is often one of the first and most important indications of cerebral disease in early life, the stomach so readily sympathising with the state of the brain. These symptoms ought always to excite our prompt attention. The secretions become depraved, the tongue foul, the bowels irregular in their action, the liver torpid, and occasionally there is a tympanitic state of the abdominal region; the appetite is capricious—sometimes entirely gone.

It is a matter of great importance to form a correct diagnosis in this complaint, for there are affections of the brain and nervous system which simulate true hydrocephalus,—we mean inflammatory hydrocephalus,—but which require for their treatment a plan essentially different from that which ought to be adopted for the cure of the more active affections of the brain. In forming our diagnosis, we must be guided by the history of the case and the consideration of the entire symptoms.

Simple acute meningitis may, in general, be early known and distinguished from the tubercular form, by closely investigating the cause and history of the disease; by observing the appearance and general health of the child we have to deal with; by noticing the violence of the attack and rapid increase of bad symptoms—viz., very acute headache, delirium, coma; by the incessant vomiting, thirst, and higher fever present, almost from the first. Constipation does not form so prominent a feature of the acute attack; the aggravation of the disorder is very quickly progressive, while in the tubercular form the course is comparatively slow, irregular, and often much prolonged.

Acute meningitis has been known to prove fatal in twenty-four hours. When it follows upon any special cause, such as eruptive fever, scarlatina, for instance, that previous occurrence will form a great aid in our diagnosis.

Meningeal apoplexy and cerebral hæmorrhage are so very rarely met with in children, that they will seldom cause us much trouble. Where any obscurity exists, we may remember in those cases the irregularity and sudden accession of symptoms, the absence of quick pulse and febrile excitement, with the early supervention of convulsions and delirium. M. Legendre attempts to found a diagnosis between cerebral hæmorrhage and simple meningitis, by noticing the presence of flexions of the thumb and great toe, or carpopedal convulsions, as they have been termed, in the former cases, and their absence in the latter. We are not able, from our own experience, to say what value attaches to this as a diagnostic mark, having never had an opportunity of examining a fatal case of cerebral hæmorrhage in an infant; but we have repeatedly seen such flexures present in cases which were considered to depend merely upon the irritation of dentition, and in which recoveries took place.

Hypertrophy of the brain, and phlebitis of the sinuses of the dura mater, are very rare occurrences also; the former disease is very slow in progress, and attended with visible enlargement of the head, from a yielding of the parietes, when it occurs in the very young child, or has made much progress; a very slight attention to, and study of the case, will reveal the true nature of the affection. We have not seen phlebitis of the sinuses, but it has been accurately described and diagnosed. When it occurs, the early symptoms are exceedingly acute, and followed soon by syncope, dilatation of the pupils, strabismus, grinding of the teeth, alternate relaxation and contraction of muscles, &c.

The diagnosis from convulsions, arising from worms and other remote causes of irritation, must be made by close attention to the history of the case, and examination of physical signs of disease elsewhere.

The convulsions which are caused by pneumonia in children, and those from spasmodic croup and spasm of the glottis, are generally accompanied by such a train of symptoms as will throw sufficient light upon their causes, and there is very little likelihood of a mistake being made as regards them.

In relation to the diagnosis of hydrocephalus and those disturbances of the brain so frequently associated with attacks of infantile remittent, Dr. V. Duke observes—"I would again warn those who may have charge of cases of remittent fever, to watch, closely and anxiously, every change; the less frequent the remissions, the greater danger would there seem to be of the invasion of hydrocephalus. Our diagnosis may be assisted by observing, in the former, those remissions occurring with regularity. The countenance, too, though expressive of suffering, is different from that in hydrocephalus; the lips are retracted or drawn, so as to show the teeth or gums; the countenance is pale, or sallow and sunk. The child seems to dread motion, lies on the back, with the knees bent or drawn up, and is pained by pressure on the abdomen. 'Here, neither the brow is knit, nor the pupil of the eye affected.' (Maunsell and Evanson.) The heat of the skin is much greater in remittent fever; the pulse, also, is quicker, and the thirst more intense; vomiting, too, is more constant. The tongue is more loaded, and sometimes is red and pointed; the evacuations are very different in remittent fever, and procured with greater ease, diarrhœa, rather than constipation, being present. The lips are generally red, chapped, and fissured, and there is often stomatitis or general abdominal tenderness. Such are the principal points of difference between the two disorders; but candour obliges me to say, that the distinction seems easier made upon paper than by the bedside of the patient. I would take this opportunity of bearing testimony to the general accuracy of, and great value to be attached to, the observations of mothers; naturally acute, their powers of observation seem heightened by parental anxiety, and they have time and opportunities for making remarks that we cannot command. Put yourself, then, in full and free communication with the mother; tell her what you suspect or want to ascertain, and direct her attention to the matters you wish elucidated. This will serve you far more than a 'dignified reserve,' and cannot injure your patient, (provided a proper time is selected for the conversation,) as might be the case were he of more mature years, and likely to be excited by being told a good deal of the doctor's opinion."

We have frequently symptoms of hydrocephalus as the result of difficult dentition. In these cases, the gums should be carefully examined and effectually lanced, and the state of the secretions particularly attended to.

When this disease is fully formed, the prognosis is unfavourable. There is a general consonance in all the symptoms of amendment, when the disease yields to treatment. In acute cases rapidly running their course, the brain and its membranes, after death, present evidences of the effects of inflammation; in other forms of the disease, the tubercular deposit will be detected. When these depositions take place in the membranes of the brain, they occur as "minute, flattened, spherical bodies, of the size of a small pin's head, or smaller, and either of a yellowish colour and rather friable under pressure, or greyish, semitransparent, and resistant, almost exactly resembling the grey granulations which are sometimes seen in the lungs or pleuræ of phthisical subjects."

In the earlier stage, these deposits look like "small opaque spots, communicating no perceptible roughness to the membranes. This appearance is absorbed in the arachnoid, covering the cerebellum and base of the brain. The fine grey bodies are mostly seen about the front, or imbedded in the pia mater, in the neighbourhood of the optic nerves.

"The surface of the brain generally presents but trifling appearance of disease, though sometimes the convolutions are flattened and the sulci partially obliterated, by the pressure of fluid contained in the ventricles.

"The appearances observed when the case has been acute, and inflammation has been decidedly present, are somewhat different. The vessels of the membranes will be found full and turgid. There is generally considerable vascularity of the pia mater; slight thickening and dulness of colour, or opacity of the arachnoid membrane, are also observed, with a dryness of that membrane, and occasionally an effusion of serum between the membranes. Lymph is also found around the vessels, and between the sulci. These changes are most apparent in the base of the brain.

"When we come to cut into the substance of the brain itself, we may find it very little altered from the natural appearance. The ventricles usually contain serum, varying from two to six ounces in quantity. The cerebral substance around the ventricles is often much softened, and breaks down very readily. Dr. West describes it as having the appearance of having been 'soaked in serum.' The quantity of fluid found in the ventricles is very variable; sometimes we find least where most had been expected, and *vice versa;* occasionally, the quantity is so large that there would seem to have been a considerable absorption of cerebral substance. In one case, where there had been considerable pain in the left side and back of the head, I found the choroid plexus occupying the inferior cornu of the lateral ventricle of that side, enlarged, and much more vascular than the corresponding one of the other side. The cerebral substance in this case, also, appeared, when cut into, dotted with bloody points, and this, too, chiefly at the left side.

"The organs in which tubercles are most frequently found, at the same time, are the lungs, bronchial glands, the spleen, liver, mesenteric glands, and intestines. 'Tubercular ulceration of the intestines,' Dr. West says, 'may give rise to diarrhœa; hence, constipation may not be an invariable symptom of hydrocephalus.'

"A morbid condition of the liver and intestinal mucous membrane has also been observed by Dr. Cheyne. He found 'the intestines inflamed and constricted, as from spasms, and the surface of the liver of a bright red colour, abounding in minute vessels, and sometimes extensively adhering to the peritoneum.' In many dissections, also, he found the surface of the liver studded with small white tubercles, not larger than grains of mustard. In every examination I have had an opportunity of making or assisting at, more or less distinct evidence was seen of the above pathological changes in the abdomen."

With regard to the treatment of this cerebral disease, Dr. Duke cautions us against "a too expectant plan, or that of treating symptoms as merely functional, or arising from some cause unconnected with the state of the brain itself, until, perhaps, fatal mischief has been done, and hydrocephalus declares itself in its most unquestionable shape."

Early attention should be paid to the state of the abdomen and alimentary canal. Dr. Duke commences his treatment by giving an emetic. He vouches for the safety and great success of this treatment. He prefers tartar emetic in the early stages, unless contra-indicated by the presence of debility. It is said to have a sedative and tranquillizing effect, and acts beneficially in lowering and equalizing the circulation, and unloading the vessels of the brain. He recommends the sparing use of purgatives. We must avoid keeping the digestive apparatus in a continual state of irritation by the exhibition of drastic or other cathartics. The head should be kept cool, and sponged with cold water. Bleeding, both local and general, must be resorted to when the symptoms are such as to indicate active disease going on in the head, and where the pulse, general temperament of the child, and other symptoms, justify us in believing the affection to be inflammatory in its nature. Great relief is sometimes obtained by the application of leeches to the Schneiderian membrane, and also to the region of the liver. Dr. Duke recommends the cautious administration of mercury combined with opium. He places this in the foremost position amongst our means of cure. He considers the fears entertained by many of the use of opium in cerebral affections exaggerated. He says it is a most valuable assistant. Dr. Duke generally recommends calomel and opium, hydrarg. c. mag. with pulv. ipecac. comp. and pulv. antimonialis. He observes—"I must speak in the highest terms of hydrargyrum cum creta or magnesia with ipecacuanha, as an alterative, not only in this, but in many other diseases of children. It seems to possess peculiar powers in restoring the healthy secretions of the mucous membrane of portions of the alimentary canal, and generally acts gently, also, upon the skin."

The early application of blisters to the nape of the neck is spoken highly of. The discharge from the blistered surface should be kept up by means of mercurial ointment with savine. We must, however, watch the effect of blisters, for frequently the irritation is so great, particularly in young children, that the cerebral disturbance is aggravated by their application. The legs and feet should have warm moist flannels applied to them. Dr. Duke does not speak favourably of iodine. He saw a case connected with the scrofulous diathesis recover under the administration of iodine combined with mercury. The form used was the iodide of potash in solution with corrosive sublimate, made into small pills with crumbs of bread. The biniodide of mercury is spoken highly of. Support should be given to the child during this course of treatment by means of broths, jellies, arrow-root, light puddings, &c.

"Stimulants may occasionally be required, but must, of course, be given with great caution. Camphor mixture, with aromatic spirit of ammonia, will often allay vomiting, and rouse the system; even when coma has set in, such stimulants have acted most beneficially; and I have seen very good effects produced by a dose of croton oil. I once sat by the bed of a child, who, to all appearance, was sinking under the effects of hydrocephalus. There had been a gradual loss of vision, and stertor and difficult breathing seemed to indicate the supervention of coma. A blister to the nape, which vesicated well, and a dose of croton oil, which acted freely, were the means which seemed to arrest the morbid action. The boy ultimately recovered.

"When the urine is scanty, which is very often the case, some authors recommend the use of squill and digitalis. I have not any personal acquaintance with their effects in those cases, having found other means sufficient; but I would say, that digitalis is not an easily managed medicine with children."

On the *prophylactic treatment* we cannot do better than quote the words of our author:—

"The state of the stomach and bowels should be particularly attended to. Children are prone to over-eat, and parents to give them too much. Many of their maladies arise from this cause, as I could quote case upon case to prove. Children should not have meat nearly so often as the generality of those in the upper and middle ranks are given it. The frequency should, in some respects, depend upon the opportunities they have for exercise, and being in the open air. Two, or even three days' abstinence in the week from solid meat would do much to preserve health, and prevent the necessity for those frequent powders so much in use. If this be thought 'diète absolue,' let them have broth on one of the abstaining days. When weather and other circumstances permit, children should spend many hours daily in the open air; even when the weather is cold, they will often enjoy being out of doors, if sufficiently clothed; and here I must enter a protest against the modern fashion of only half clothing children. I would rather my child wanted a meal, than see it subjected to that daily starvation from cold, to which those poor little fashionables, who trudge along with measured step, are exposed, as to their lower limbs especially.

"Too little attention is generally paid to regularity of hours for giving children their meals; when practicable, an early dinner hour should be fixed on for them. Nothing requires more care than their sleeping apartment. Enter an ordinary nursery between eight o'clock at night and the same hour in the morning. If the occupants be numerous, you find it generally very close, and ill-ventilated,—every child's bed, perhaps, furnished with its own nice curtain, and placed in the closest corner that can be selected. Here they spend half their entire time, hour after hour, inhaling a vitiated atmosphere, and slowly sowing the seeds of future disease. Let the room door be left open at night, and have a ventilator placed high up near the ceiling, opening into the flue; a hole broken will answer every purpose. Let curtains be banished from the nursery, and all useless furniture removed, and at times that the children are absent, let the windows be opened for thorough ventilation. In addition, let daily ablution be practised with every child; it keeps the skin in a healthy state, and very much lessens the liability to disease.

"Where the tendency to hydrocephalus exhibits itself very strongly, either in a constitution hereditarily delicate or acquired, I would recommend the formation of an issue in the arm; and where the disease has been unequivocally present, I have seen the best effects follow an issue established at the top of the head."

On the subject of chronic hydrocephalus, Dr. Duke says—

"A child may be, apparently, born healthy, and for the first few weeks or months not attract any particular attention. The earliest symptoms which we notice of incipient disease are, generally, a wasting of flesh, and a want of thriving appearance. The child loses its plumpness, and becomes soft, and this, although the appetite may be good; sometimes the appetite is even craving, and there is a disordered condition of the bowels; they are constipated, or irregularly free. Convulsions may take place very early and frequently, and cannot be traced to any particular cause. A rolling of the head and eyes, and, perhaps, strabismus, may exist, and then, soon, an enlargement of the head will be noticed. This increases very gradually, but continuously, and is accompanied with tension of the fontanelles, and fulness of the veins of the forehead and temples. The head becomes so large and heavy, that the child is unable to support the weight of it, and it hangs to one side. In the very young child, the enlargement of the head proceeds rapidly, and to a great extent, from the ready yielding of the parietes. The disease is almost invariably fatal. Some cases are considerably protracted, and a life of misery is terminated by convulsions, or seeming exhaustion. Occasionally, acute inflammation sets in, and proves the immediate cause of dissolution.

"Dissection shows, that in some cases the fluid is contained in the sac of the arachnoid, constituting what is termed external hydrocephalus, whilst in the vast majority of cases the lateral ventricles form the seat of the collection. The quantity of this fluid varies, of course, with the persistence of the disease, and the size the head may have attained. Sometimes it presents the appearance of pure limpid serum; at others, especially when symptoms of inflammation have been present long before death, it appears turbid, with flocculi of lymph in it.

"The lining membrane of the ventricles becomes changed. Dr. West says—'Even when no false membrane is formed within the ventricles, their lining often presents other evidence, besides mere thickening, of its having been the seat of inflammation: it is roughened, and granular, presenting an appearance closely resembling shagreen, and communicating a very perceptible roughness to the finger. All parts do not seem equally liable to undergo this change; but I have observed it to be much more marked about the corpora striata than elsewhere.'

"Regarding the mode of treatment, very little can be said in favour of the success of any particular plan. Some cases are decidedly and hopelessly incurable. In these, very frequently recurring convulsions and paralysis will be present. Those in which there is some likelihood of cure are characterized by simple enlargement of the head, without much convulsive action.

"Gölis recommends, 'that the hair be kept closely cut, and that one or two drachms of mild mercurial ointment be rubbed daily into the scalp; at the same time, from a quarter to half a grain of calomel should be given twice a-day, unless diarrhœa come on, when the inunction alone must be performed.'

"Blisters to the nape, occasional leeching, if there be much heat of head, and the administration of a purgative, now and then, seem to be the chief means upon which any reliance can be placed. A couple of small issues in the back of the neck may also be of service, by keeping up a long-continued drain.

"Mr. Barnard, of Bath, has recommended, and spoken highly of the success attending a mode of bandaging the head, so as to make pressure upon the parietes of the cranium, and prevent their further expansion.

"Puncture of the cranium, and evacuation of the fluid collected, have also been practised. The extreme difficulty of determining, with any degree of accuracy, the situation of this fluid, whether within the ventricles or external to them, must form an insuperable objection to the adoption of this means; and, unfortunately, the recorded results of cases thus treated do not justify its general recommendation.

"On the whole, chronic hydrocephalus is one of that class of diseases which it is so very painful to the physician to be occasionally obliged to witness through their various stages. He can scarcely either give or indulge in hope of a favourable termination, and sometimes, even when apparent recovery may take place, the intellect will remain in a permanently weakened condition."

With this extract we conclude our notice of Dr. V. Duke's prize essay "*On the Cerebral Affections of Infancy and Childhood.*" We have derived much pleasure, and we trust profit, from its perusal.

We have endeavoured to give our readers a faithful analysis of the author's views; and although he has advanced nothing new with reference to the therapeutics or pathology of this class of affections, his essay may be considered as a correct outline of the present state of science in relation to this department of practical medicine.

